# Effects of Cognitive/Exercise Dual-Task Program on the Cognitive Function, Health Status, Depression, and Life Satisfaction of the Elderly Living in the Community

**DOI:** 10.3390/ijerph18157848

**Published:** 2021-07-24

**Authors:** Sohyune Sok, Eunyoung Shin, Seyoon Kim, Myeongshin Kim

**Affiliations:** 1 College of Nursing Science, Kyung Hee University, Seoul 02447, Korea; 2 Department of Nursing, Graduate School, Kyung Hee University, Seoul 02447, Korea; eyshin@khu.ac.kr (E.S.); ssamyun85@naver.com (S.K.); myeongshin@khu.ac.kr (M.K.)

**Keywords:** aged, cognition, exercise, dual-task, community

## Abstract

The elderly population in Korea is rapidly increasing. It is necessary to develop multi-faceted and complex interventions for prevention and delay of dementia, balance improvement, and physical activity, among the elderly living in the community. This study aimed to examine the effects of the cognitive/exercise dual-task program on cognitive function, health status, depression, and life satisfaction of the elderly living in the community. A quasi-experimental study design using a pretest-posttest control group was employed. The study included a total of 65 elderly participants (intervention: *n* = 32, control: *n* = 33) in Seoul, South Korea. The cognitive/exercise dual-task program as an intervention was composed of 20 sessions for a total of 10 weeks, held twice a week for about 50 min each session. Measures were general characteristics of study participants, the Korean version of Mini-Mental State Examination (MMSE-K), Korean elderly health status assessment tool, Korean version of Geriatric Depression Scale, and the elderly life satisfaction scale. Data were collected from October 2020 to March 2021. There were statistically significant differences on cognitive function, health status, depression, and life satisfaction between two groups. The cognitive/exercise dual-task program was an effective intervention for improving cognitive function, health status, and life satisfaction, and for decreasing depression of the elderly living in the community. Health care providers need to pay attention to cognitive/exercise dual-task programs for elderly living in the community.

## 1. Introduction

Korea is expected to enter an ultra-aged society, as the ratio of the elderly population aged 65 years or older was 15.7% in 2020 and is estimated to be 20.3% by 2025 [1]. The number of patients with dementia rapidly increased, along with the elderly population. The cost of managing dementia was KRW 16,331.6 billion in 2019, an increase of KRW 640.7 billion from KRW 15,690.9 billion in 2018 [2]. As a countermeasure, the government introduced a national dementia responsibility system in 2017, opened dementia relief centers nationwide, and began operating various services related to dementia by switching to customized case management [3].

As the elderly age, their subjective or objective memory is significantly reduced, and they experience difficulties in daily life activities and social life [4,5]. Similar to Alzheimer’s disease, it was reported that not only decreased cognitive function, but also problems of parietal and temporal lobe areas of the brain are shown in simple perceptual tasks, thereby resulting in decreased spatio-temporal compositional ability and motor perception problems [5,6]. In addition, it was found that with decreased cognitive function, muscle mass and muscle strength also decreased [4]. Muscle mass is the main factor influencing muscle strength, and loss of muscle mass due to aging leads to muscle weakness [7]. Muscle strength is further lowered by the reduced physical activity of the elderly, thus resulting in poor physical health, which affects their emotional and social health status [7,8].

Moreover, depression as a mental problem for the elderly is not a disease caused by aging, but it is a common mental health problem that can be experienced in old age. However, depression is a serious disease that can lead to thoughts about giving up on life. It is closely related to the quality of life for the elderly, and it can also lead to a decrease in the state of physical health and mental cognitive function [9]. According to previous papers, the physical activity program of the elderly is closely related to depression, and it has meaning in physical activity itself in regard to lowering the degree of depression by helping them gain confidence and achieve physical efficacy by moving the body [3]. The elderly’s depression has an effect on their life satisfaction, so it is important to measure and improve life satisfaction, and lower the degree of depression [10,11]. The life satisfaction of the elderly refers to a subjective measure of the degree of satisfaction with an optimistic expectation of various realistic variables that can affect the psychological changes in one’s life [10]. The life satisfaction level of adults in Korea is 5.9, which is lower than the OECD average of 6.5 (out of 10 points), thus indicating that the level of life satisfaction is low [12]. It also shows that life satisfaction continues to decrease as the age increases [13]. In addition, as the suicide rate and poverty rate in old age are higher than in other life cycles [11], it is an urgent task to increase the life satisfaction of the elderly. The elderly’s life satisfaction was said to have a positive effect on participation in sports or exercise [14].

Based on the results of the previous studies, various nonpharmacological interventions have been attempted to improve the cognitive and physical conditions of the elderly and manage the progression of dementia [2,4,5,6,9,14,15,16]. In particular, cognitive or exercise programs are being developed in various disciplines, such as medicine, psychology, rehabilitation, and sports [2,6,7,8,13,15]. However, most of them were single-task interventions, and the subjects were the elderly in the community, or the elderly admitted to nursing homes. Moreover, criteria, such as the type or number of programs, were not clear. As the effect of cognitive or exercise therapy, developed as a single program, was partially evaluated for the characteristics of the elderly, the effect may be limited or insufficient if used continuously [17].

The cognitive programs that have been proven to be effective for the elderly are reminiscence therapy and memory training programs, which have been found to be effective mainly on cognitive function, depression, and some of memory capacity [18,19]. In particular, photo cards used in the cognitive programs improved cognitive function and memory capacity [18,19].

In the exercise program, fumanet, or square stepping exercise (SSE) is to perform a fumanet task stepping one by one, and this was effective in balance, gait, lower extremity muscle strength, memory recall, and attention in the elderly with dementia or mild cognitive impairment [2,16,20]. Square stepping exercise comprises multiple directional step patterns performed on a thin mat which is partitioned into squares 25 cm^2^ each, and may include toe walking or complex pattern of walking as a progression. SSE can also be performed indoors, so certainly appears to be advantageous over unidirectional and outdoor walking, which is less beneficial pertaining to fall prevention, and it is also unsafe for older adults [21,22]. Square stepping exercise strengthens the quadriceps and hamstring muscles of both legs and also improves balance [22,23,24]. However, no improvement in cognitive function was found in previous studies [22,23,24], and although there are the effects of cognitive and psycho-emotional well-being, safety issues have been raised when the exercise is applied to the elderly [21].

Physical-cognitive dual-task interventions for older people have provided evidence of efficacy to improve both physical health (lower extremity muscle strength, etc.) and cognitive function (especially executive function) [25,26,27,28,29]. Fumanet, having the characteristics of physical-cognitive dual-task training, may also elicit physical and cognitive health gains jointly [2,20]. Then, it is necessary to develop multi-faceted and complex interventions that improve health status, reduce depression, and improve life satisfaction, such as prevention and delay of dementia, balance improvement, and physical activity promotion, among the healthy elderly living in the community.

Fumanet exercise, the cognitive/exercise dual-task program used in this study, offers the benefit of improving both cognitive and physical functions [2,20]. The duration and frequency of application of this program to obtain benefits was 8 to 12 weeks, twice a week, and 40 to 50 min per session in previous studies [3,20,25,27].

It has been reported in previous studies that the cognitive/exercise dual-task intervention is more effective for both physical and cognitive functions, not only for the elderly with mild cognitive impairment or dementia, but also for the healthy elderly [2,3,21,25,26,27,28,29]. However, in terms of preventing dementia, it is more important to apply the intervention to the healthy elderly [26,27,29]. Therefore, this study on implementing the intervention to healthy elderly is worth conducting and can be considered a novel application. It intends to apply the cognitive/exercise dual-task program for dementia prevention and physical health improvement in healthy elderly living in the community and verify its effectiveness.

The interaction model of Cox [30], which changes the subject’s health behavior and derives positive health outcomes, is an intervention model that explains the subject’s change through the subject–expert interactive factor. It is suitable for application to nursing intervention programs applicable to the elderly living in the community, as the subject can have an interactive factor with the expert by actively participating in the nursing intervention provided, and strengthen health promotion behavior via motivation. When the cognitive/exercise dual-task program was started, a demonstration of dual-task training by the researcher (expert) was firstly presented for the study participant. After that, the intervention through the study participant–researcher interaction was proceeded on each step. The interaction successfully resulted in positive health outcomes and, by the last step, with the improved health behavior of study participant.

**Figure 1:**
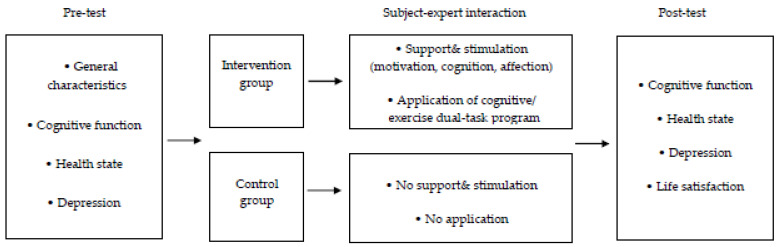
Conceptual framework of this study.

Based on previous studies [2,14,17,20,27,28], this study performed a dual-task program that combined reminiscence therapy, memory training program, and fumanet exercise in order to perform cognitive therapy and exercise therapy at the same time. Using the subject–expert interactive factors of Cox [30] as an intervention strategy, a comprehensive assessment on cognitive function, health status, depression, and life satisfaction of the elderly living in the community was performed (Figure 1). The objectives of this study were to verify the effectiveness of the cognitive/exercise dual-task program on cognitive function, health status, depression, and life satisfaction of the elderly living in the community.

## 2. Material and Methods

### 2.1. Study Design and Participants

A quasi-experimental study design using a pretest-posttest control group was employed. The study participants of this study are the elderly, aged 65 years or older, residing in Seoul, who can communicate and read the Korean, have a cognitive function score (MMSE) of 21 or more, are not currently acutely ill, can be living daily in the community, and have agreed to participate in this study. They did not receive drug treatment related to the improvement of cognitive function and depression, and they have not participated in cognitive or exercise programs conducted by other institutions. The study participants were selected through convenient sampling and randomly assigned to the intervention group or the control group using a coin toss.

In this study, when the effect size was 0.5, the significance level was 0.05, and the desired statistical power level was 0.8, using the table of statistical power analysis for the behavioral sciences presented by Cohen [31], the intervention group and the control group included 31 participants each. The total number of study participants was 65, with 32 in the intervention group and 33 in the control group.

### 2.2. Experimental Intervention

The cognitive/exercise dual-task program was developed by using Cox’s interaction model [30], which changes the subject’s health behavior through the subject–expert interactive factor and derives positive health outcomes. In addition, the program was verified by an expert group consisting of 1 professor of rehabilitation medicine, 1 professor of geriatric internal medicine, 1 geriatric nurse specialist, and 3 head nurses in the geriatric ward for the validity of contents and process of the cognitive/exercise dual-task program.

Experimental intervention for the humanet exercise, the cognitive/exercise dual-task program, was carried out with the following procedure.

After connecting two nets with a combination of rubber straps, in 3 horizontal and 4 vertical rows in a square about 50 cm in size, to make 3 horizontal and 8 vertical rows, the participants were instructed to slowly walk with various steps using squares while taking care not to step on the net. Songs and rhythms were added to the exercise to promote a sense of recreation. The program was conducted twice a week, 50 min per session, and the 32 subjects in the intervention group were divided into 2 small groups. A total of 20 sessions were provided for a total of 10 weeks. One researcher was the instructor for each session, and after checking the physical condition and safety of all participants, they were made to sit on a chair and move their feet to relax and stretch before starting the exercise. After performing the basic three steps, No. 1, 2, and 3, once, task step No. 4 and task step No. 5 were sequentially performed. All steps were performed in one direction with the instructor, and while one person walks on the net, the other participants sit in chairs and follow the steps until it is their turn. In task step 5, participants were asked to follow the steps and songs together. The participants were asked to drink water after they finished the step. After checking their physical condition, they were asked to look at the photos used during the basic and task steps again and share their thoughts and feelings. The components of session one were repeated every session.

**Figure 2:**
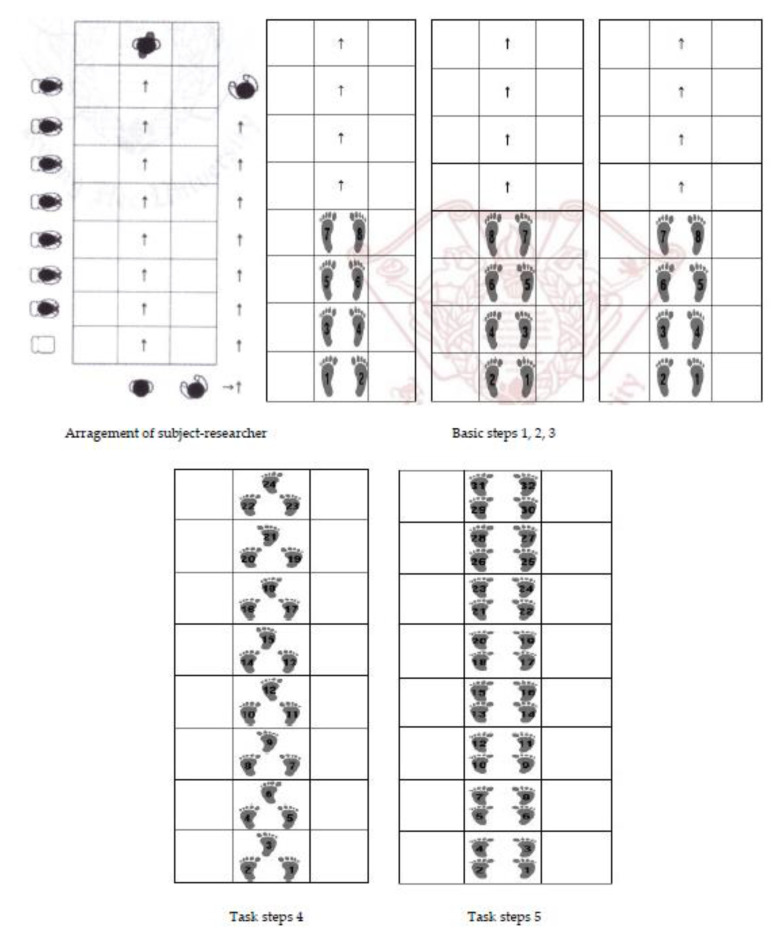
The basic and task steps of humanet exercise.

As for the rationale for the duration and method of the program, the result showed that the index improved when an 8 or 10-week training program was applied to the elderly in the previous studies [2,20,32]. The program was conducted as a group training, with the researcher performing a demonstration in front of the participants, who then followed. The composition of the program is shown in Table 1, and the basic and task steps of the humanet exercise presented in Figure 2. The task steps of humanet exercise were more difficult, complex, and higher levels than basic steps.

### 2.3. Measures

The general characteristics of the study participants consisted of gender, age, education, marital status, living with, religion, chronic disease, drinking, smoking, exercise, and participation in dementia prevention program. This consisted of a total of eleven items.

Cognitive function was measured by using the Korean version of Mini-Mental State Examination (MMSE-K), which was revised by Kweon and Park [33] for the Mini-Mental State Examination (MMSE) developed by Folstein, Folstein, and McHugh [34]. The MMSE-K is a cognitive function scale that evaluates the orientation, attention, calculation ability, language function, and judgment ability of the elderly in Korea. It consists of 5 dimensions with 12 items. The range of the score is from 0 to 30, and the higher the score, the higher the degree of cognitive function. In the study of Kweon and Park [33], Cronbach’s α = 0.92, and in this study, Cronbach’s α = 0.94.

The Korean elderly health status assessment tool developed by Kim et al. [35] was used. This tool consists of 24 items in 3 dimensions: physical dimension (15 items), emotional dimension (5 items), and social dimension (4 items). With a 4-point Likert scale, it has a range of scores from 24 to 96 points, and the higher the score, the better the health of the elderly. The reliability in this study was Cronbach’s α = 0.86.

The Korean version of Geriatric Depression Scale (GDS) developed by Sheikh and Yesavage [36] and verified by Ki [37] was used. This scale consists of 15 items. If the answer is ‘yes’, it is converted to 0 point, and if the answer is ‘no’, it is converted to 1 point. However, the negative 10 items were calculated reversely. The total score ranged from 0 to 15, with a total score of 1 to 5 indicating a normal state, and a score of 6 to 15 indicating a depressed state. The higher the score, the more severe the degree of depression. The reliability in this study was Cronbach’s α = 0.87.

As an instrument for measuring life satisfaction, the elderly life satisfaction scale standardized by Choi [38] for the elderly in Korea was used. This scale consists of 20 items in total: satisfaction related to the past (6 items), satisfaction related to the present (9 items), and satisfaction related to the future (5 items). A 3-point scale was used, and the range of scores was from 20 to 60 points. The higher the score, the higher the satisfaction with life. The reliability of this instrument was Cronbach’s α = 0.90 at the time of development. The reliability in this study was Cronbach’s α = 0.89.

### 2.4. Procedures

This study was conducted from October 2020 to March 2021. The cognitive/exercise dual-task program for the elderly living in the community was a program consisting of 20 sessions for 10 weeks, and it was conducted through training and education at each session. The experimental intervention was directly applied by the researcher to the intervention group. The researcher is a geriatric nurse specialist with more than 3 years of teaching experience at a university. The program was held in a community center for senior citizens. In the preliminary survey, general characteristics, cognitive function, health status, depression, and life satisfaction were surveyed with a questionnaire for all study participants in the intervention and control groups. The post-survey was conducted immediately after the end of the program in the intervention group. In the control group, at 10 weeks after the preliminary survey, it was conducted in the same manner as the preliminary survey. During the 10 weeks, participants in the control group were living daily in their community. After the 10 weeks, cognitive function, health status, depression, and life satisfaction were measured for all study participants in the intervention and control groups. At this time, it was conducted by the same research assistant who conducted the preliminary survey. After the study finished, the same cognitive/exercise dual-task program was provided for the participants in the control group as an ethical consideration.

### 2.5. Statistical Analysis

The data were analyzed as follows using the SPSS/WIN 21.0 program (IBM, Armonk, NY, USA). First, the general characteristics of the study participants were analyzed using descriptive statistics. Second, the homogeneity test for, and difference in, the general characteristics of the study participants, and study variables between the intervention group and the control group, were analyzed using the chi-square test, Fisher’s exact test, and independent t-test. Third, the effects of the cognitive/exercise dual-task program were verified by using the independent t-test.

### 2.6. Ethical Considerations

This study was conducted after obtaining approval from the K University Institutional Review Board (KHSIRB-20-193 (RA)). We visited the senior citizen’s center and the relevant ward office in advance to explain the purpose and procedure of the study to the facility director. After obtaining approval, the recruitment guide was distributed to the facility to explain the purpose of the study. Following the explanation of the purpose and procedure of the study, a written consent was obtained from the elderly, who decided to voluntarily participate. In the informed consent form, the purpose of the study, the operation method of the program, the procedure of data collection, and the benefits and discomfort that could be obtained from participating in the study were explained, as well as the compensation to the subject. It was explained that the informed consent form would be strictly confidential in principle of anonymity, and that the study participants can withdraw at any time if they did not want voluntary participation.

## 3. Results

### 3.1. General Characteristics of the Study Participants and Homogeneity

Table 2 shows the general characteristics of study participants and homogeneity. In the general characteristics of the study participants, there were more females than males, 62.5% in the intervention group and 60.6% in the control group. In terms of age, the average age of the study participants was 73.78 years (73.66 years in the intervention group and 73.91 years in the control group). In terms of marital status, more than half of the study participants were married (56.2% in the intervention group and 57.6% in the control group). In terms of cohabitants, the majority of the study participants were living alone (40.6% in the intervention group and 42.4% in the control group). In terms of frequency of chronic disease, having one chronic disease was the highest (43.8% in the intervention group and 51.5% in the control group). All of the study participants were drinking, and there were slightly more nonsmokers than smokers (59.4% in the intervention group and 51.5% in the control group). In terms of exercise, the majority of the study participants exercised sometimes (62.5% in the intervention group and 54.5% in the control group). In the general characteristics of the study participants, the intervention group and the control group were statistically significantly homogeneous at the significance level *p* < 0.05 (Table 2).

### 3.2. Homogeneity in Study Variables before the Experimental Intervention

Table 3 shows the homogeneity in the study variables before the experimental intervention. Variance between the intervention and the control group were all non-significant, ensuring homogeneity between the two groups at the significance level *p* < 0.05 (Table 3).

### 3.3. Effects of Cognitive/Exercise Dual-Task Program

Table 4 shows the application effect of the cognitive/exercise dual-task program. The cognitive/exercise dual-task program statistically significantly improved cognitive function (t = 7.349, *p* < 0.001), health status (t = 16.405, *p* < 0.001), and life satisfaction (t = 5.411, *p* < 0.001) of the elderly living in the community, while significantly decreasing depression (t = −4.400, *p* < 0.001) (Table 4).

## 4. Discussion

This study analyzed the effectiveness of the program after applying the cognitive/exercise dual-task program for the elderly living in the community. As a result of the study, the cognitive function, health status, depression, and life satisfaction of the elderly who participated in the cognition/exercise dual-task program were significantly improved than those of the elderly who did not.

In this study, there was a statistically significant improvement of about 20% in cognitive function as a result of the intervention. In the cognitive/exercise dual-task program, the basic steps and task steps of fumanet were sequentially followed, and a photo card was placed on the 4th and 8th spaces, to which the subjects were tasked to guess the answer within 5 s after showing it. Sharing opinions while seeing the photo card has been shown to improve the cognitive function of the elderly. As cognitive function is an important criterion for diagnosing dementia [4,15], it was confirmed that the cognitive/exercise dual-task program improves cognitive function and helps in the prevention of dementia. This was consistent with the results of the previous studies reporting that exercise programs for the elderly had a positive effect on cognitive function or dementia prevention [5,6,9,15].

In the intervention group, health status was significantly improved after participating in the cognitive/exercise dual-task program. This was consistent with the results of previous studies showing that various exercise programs were effective on the health status of the elderly [14,15,32]. In this study’s cognitive/exercise dual-task program, it was confirmed that performing fumanet exercise with friends and sharing opinions using photo cards resulted in the health status improvement of the elderly living in the community.

The result of this study showed that the degree of depression of the elderly in the intervention group significantly decreased after applying the cognitive/exercise dual-task program. Among the previous studies related to this, a study that confirmed the effect size after the exercise program also reported that the exercise program had a great effect of −0.910 in alleviating depression in the elderly [39]. In addition, previous studies that applied yoga, stretching, resistance exercise, and health promotion exercise program to the elderly reported that such exercise programs significantly reduced depression [5,9,40,41,42], which was consistent with the results of this study.

In this study, it was found that life satisfaction was significantly improved in the intervention group after applying the cognitive/exercise dual-task program. It was similar to the results of the previous studies that reported improvement in life satisfaction after applying the exercise program, physical activity, pilates exercise, and occupational therapy exercise for the elderly [9,13,42]. This supports the results of this study by showing that exercise programs have a positive effect on the life satisfaction of the elderly. In addition, a previous study showed that life satisfaction was increased by performing exercise therapy for the elderly with low back pain [14]. Life satisfaction is a subjective feeling, and as the pain or stress chronically experienced by the elderly living in the community is reduced, satisfaction can increase. In this cognitive/exercise dual-task program, it is inferred that stretching, walking in place, and shaking legs from warm-up and finishing exercises relieved joint stiffness, pain, and stress, and fumanet step exercise and opinion sharing caused mood changes, interest, and fun through interaction among the elderly participants and researchers. The cognitive/exercise dual-task program used in this study was found to have a positive effect on improving the life satisfaction of the elderly living in the community.

Based on the results of this study, the cognitive/exercise dual-task program targeted the elderly living in the community and improved their cognitive function, health status, depression, and life satisfaction. Furthermore, in Cox’s subject–expert interaction model [30], which is the basis for the development of the cognitive/exercise dual-task program, the interaction between the study participants and the researcher can be said to have an effect on improving the study participant’s cognitive function, health status, depression, and life satisfaction.

In this study, based on previous studies, a dual-task program was developed by using the subject-expert interactive factors of Cox [30] as an intervention strategy, which combined reminiscence therapy, memory training program, and fumanet exercise to perform cognitive therapy and exercise therapy at the same time. This study has its significance in that the cognitive/exercise dual-task program has been applied to the elderly living in the community, and it has improved their cognitive function, health status, depression, and life satisfaction. Therefore, the cognitive/exercise dual-task program is expected to be actively used as an intervention method to improve cognitive function, health status, depression, and life satisfaction, to prevent dementia, and to promote the health of the elderly living in the community. The novelty compared to the previous studies might be the better cognitive function, better physical health, and generalizability of the efficacy of the intervention to healthy aging individuals.

As a limitation of this study, as the cognitive/exercise dual-task program is a group exercise, not an individual exercise, and the elderly met twice a week, it is necessary to confirm whether it is the result of regular contact with neighbors and peer groups sociologically. Furthermore, it has a limitation in that the elderly could not be completely controlled, i.e., we did not prevent them from performing other exercises, or from making special changes in their lives during the 10-week program.

A long-term longitudinal study to confirm the duration of the effect of the cognitive/exercise dual-task program is deemed necessary in future studies. Furthermore, in order to verify the possibility of expanding the scope of practical application of this program, it is necessary to attempt replication studies not only in various regions, but also in the elderly in other countries.

## 5. Conclusions

This study conducted a cognitive/exercise dual-task program twice a week for 10 weeks for the elderly living in the community and verified its effectiveness. As a result of the study, it was confirmed that the cognitive/exercise dual-task program is an effective intervention program that improves cognitive function, health status, and life satisfaction, and reduces depression of the elderly living in the community. Therefore, it is strongly recommended to use the cognitive/exercise dual-task program developed in this study in the community.

## Figures and Tables

**Table 1:** The composition of cognitive/exercise dual-task program.

Stage	Composition	Time
	Demonstration of dual-task training	
Warm-up exercise	Stretching, walking in place	5 min
	-Sit on a chair, and stretch neck, shoulders, flanks, and legs. -Sit on a chair, move arms back and forth, and move legs to walk in place.	
Main training	Basic step: Fumanet exercise, dual-task training (weeks 1–5)	30 min
	-Perform fumanet basic steps 1, 2, and 3 once, one by one. -All of the elderly are requested to perform fumanet basic steps 1, 2, and 3, one by one, and guess the answer for the photo cards on the box in the 4th and 8th spaces of the net within 5 s. (It does not matter if they cannot answer it.)	
	Task step: Fumanet exercise, dual-task training (weeks 6–10)	30 min
	-Perform fumanet task steps 4 and 5 once, one by one. -All of the elderly are requested to perform fumanet task steps 4 and 5, one by one, and guess the answer for the photo cards on the box in the 4th and 8th spaces of the net within 5 s. (It does not matter if they cannot answer it.)	
Finishing exercise	Stretching, shaking legs	5 min
	-Sit on a chair, and stretch neck, shoulders, flanks, and legs. -Sit on a chair and shake legs and ankles to release tense muscles and joints.	
Communication time	Sharing opinions	10 min
	-The elderly are requested to say their opinions while looking at the photo cards used during the main training.	

**Table 2:** General characteristics of the study participants and homogeneity.

Characteristics	Intervention Group (*N* = 32) *n* (%)	Control Group (*N* = 33) *n* (%)	t/χ^2^ (*p)*
Gender			
Male	12 (37.5)	13 (39.4)	0.025 (0.875)
Female	20 (62.5)	20 (60.6)	
Age (year)			
65~69	9 (28.1)	9 (27.3)	0.187 (0.980)
70~74	9 (28.1)	8 (24.2)	
75~79	9 (28.1)	10 (30.3)	
80≤	5 (15.7)	6 (18.2)	
Mean ± SD	73.66 ± 5.92	73.91 ± 6.09	−0.170 (0.866)
Mean ± SD	73.78 ± 5.96		
Education			
None	8 (25.0)	10 (30.3)	2.078 (0.556)
Elementary school	5 (15.6)	6 (18.2)	
Middle school	14 (43.8)	9 (27.3)	
High school	5 (15.6)	8 (24.2)	
Marital status			
Married	18 (56.2)	19 (57.6)	0.012 (0.914)
Bereavement	14 (43.8)	14 (42.4)	
Living with			
Alone	13 (40.6)	14 (42.4)	0.139 † (0.987)
Spouse	8 (25.0)	9 (27.3)	
Spouse, child(ren)	9 (28.1)	8 (24.2)	
Child(ren)	2 (6.3)	2 (6.1)	
Religion			
Protestant	14 (43.8)	18 (54.5)	1.303 † (0.138)
Catholic	11 (34.4)	11 (33.3)	
Buddhist	7 (21.8)	4 (12.2)	
Chronic disease			
(frequency)			
1	14 (43.8)	17 (51.5)	0.497 † (0.919)
2	6 (18.8)	6 (18.2)	
3	10 (31.3)	8 (24.2)	
4	2 (6.3)	2 (6.1)	
Drinking			
Yes	32 (100.0)	33 (100.0)	0.000 (1.000)
No	0 (0.0)	0 (0.0)	
Smoking			
Yes	13 (40.6)	16 (48.5)	0.406 (0.524)
No	19 (59.4)	17 (51.5)	
Exercise			
Regular	12 (37.5)	15 (45.5)	0.423 (0.515)
Sometimes	20 (62.5)	18 (54.5)	
Participation in dementia			
prevention program			
Yes	11 (34.4)	9 (27.3)	0.385 (0.535)
No	21 (65.6)	24 (72.7)	

† Fisher exact test.

**Table 3:** Homogeneity in study variables before experimental intervention.

Variables	Intervention Group (*N* = 32) Mean ± SD	Control Group (*N* = 33) Mean ± SD	t (*p)*
Cognitive function	21.50 ± 2.10	21.39 ± 1.89	0.215 (0.831)
Health state	62.78 ± 5.63	62.36 ± 5.82	0.294 (0.770)
Depression	10.34 ± 2.49	10.97 ± 5.23	−0.613 (0.542)
Life satisfaction	44.31 ± 5.35	44.27 ± 5.37	0.030 (0.976)

**Table 4:** Effects of cognition/exercise dual-task program.

Variables	Intervention Group (*N* = 32)			Control Group (*N* = 33)			t *p*
	Pre Mean ± SD	Post Mean ± SD	Difference Mean ± SD	Pre Mean ± SD	Post Mean ± SD	Difference Mean ± SD	
Cognitive function	21.50 ± 2.10	26.28 ± 3.40	4.78 ± 3.07	21.39 ± 1.89	21.12 ± 1.95	−0.27 ± 2.45	7.349 <0.001 *
Health status	62.78 ± 5.63	86.91 ± 4.42	24.13 ± 3.63	62.36 ± 5.82	62.52 ± 5.75	0.16 ± 2.45	16.405 <0.001 *
Depression	10.34 ± 2.49	3.50 ± 3.48	−6.84 ± 4.02	10.97 ± 5.23	9.91 ± 5.16	−1.06 ± 2.29	−4.400 <0.001 *
Life satisfaction	44.31 ± 5.35	52.09 ± 3.65	7.78 ± 5.29	44.27 ± 5.37	44.64 ± 5.27	0.37 ± 1.74	5.411 <0.001 *

* *p* < 0.05.

## Data Availability

No new data were created or analyzed in this study. Data sharing is not applicable to this article.
